# Soluble Programmed Death-Ligand 1 (sPD-L1) as a Promising Marker for Head and Neck Squamous Cell Carcinoma: Correlations With Clinical and Demographic Characteristics

**DOI:** 10.7759/cureus.44338

**Published:** 2023-08-29

**Authors:** Amani A Alrehaili, Amal F Gharib, Abdulraheem Almalki, Ahmed Alghamdi, Nahed M Hawsawi, Maha M Bakhuraysah, Hayaa M Alhuthali, Rasha L Etewa, Wael H Elsawy

**Affiliations:** 1 Department of Clinical Laboratory Sciences, College of Applied Medical Sciences, Taif University, Taif, SAU; 2 Department of Pathology, College of Medicine, Jouf University, Sakaka, SAU; 3 Department of Clinical Oncology, Faculty of Medicine, Zagazig University, Zagazig, EGY

**Keywords:** radiation therapy, chemotherapy, prognostic, biomarker, spd-l1, hnscc, head and neck squamous cell carcinoma

## Abstract

Background and objective

Head and neck squamous cell carcinoma (HNSCC) is a prevalent cancer type that affects the mucosal lining of the upper aerodigestive tract. Soluble programmed death-ligand 1 (sPD-L1) is a significant factor in hindering T cells' function, which prevents cancer cells from being detected by the immune system. This means that sPD-L1 is an essential component in the immune evasion of cancer. This study aimed to explore the potential of sPD-L1 as a prognostic biomarker for patients with HNSCC undergoing concurrent chemotherapy and radiation therapy.

Methodology

The study included 106 patients with locally advanced HNSCC who received three courses of induction chemotherapy followed by concurrent chemoradiation and 60 healthy subjects as controls. sPD-L1 levels were measured using an enzyme-linked immunosorbent assay (ELISA) kit, and the cutoff value was determined based on receiver operating characteristic (ROC) curve analysis.

Results

The results showed that sPD-L1 levels were significantly higher in HNSCC patients compared to healthy controls, with a cutoff value of 31.51 pg/mL. Higher sPD-L1 levels were associated with poorer overall survival rates.

Conclusions

These findings suggest that sPD-L1 may serve as a valuable prognostic biomarker for HNSCC patients undergoing concurrent chemotherapy and radiation therapy. The study highlights the importance of exploring new biomarkers and therapeutic strategies for HNSCC to improve patient outcomes and reduce morbidity and mortality rates associated with this disease.

## Introduction

Head and neck squamous cell carcinoma (HNSCC) is a type of cancer that most commonly affects the mucosal lining of the upper aerodigestive tract, including the oral cavity, pharynx, and larynx. Annually, there are 890,000 new cases of HNSCC and 450,000 deaths, making it a major public health issue [1]. The incidence of HNSCC varies significantly across the globe. The use of tobacco and alcohol, human papillomavirus (HPV) infection, and exposure to certain occupational and environmental carcinogens are all factors that can increase the likelihood of developing this disease [2]. HNSCC is usually identified and treated using a multidisciplinary strategy that includes surgery, radiation therapy, and chemotherapy. Treatment is determined by many criteria, including the tumor's location and extent, the stage of the disease, and the patient's overall health and preferences. In recent years, there has also been a rise in concern about the use of immune checkpoint inhibitors, which target the programmed death-ligand 1 (PD-L1) and other immune-suppressing proteins, as therapeutic options for HNSCC [3]. The PD-1 protein plays a role in regulating the immune system as it is present on the surface of T-cells, B-cells, and natural killer cells [4]. When PD-1 binds with its ligand, PD-L1, it sends a signal that suppresses T-cell activity. This helps in protecting healthy cells from T-cell attack (autoimmune reaction) [5,6]. However, many cancer cells overexpress PD-L1 to avoid the immune system's responses [7]. This enables cancer cells to grow and spread without disruptive immune system interference [8]. According to the study of Dong et al, cancer cells expressing PD-L1 can induce apoptosis in activated T-cells and prompt circulating T-cells to produce IL-10, resulting in a suppressed immune response [9]. Apart from its ability to induce T-cell apoptosis and interleukin-10 (IL-10) production, PD-L1 also has various impacts on T-cell activity [10]. First, the binding to PD-1 on T-cells inhibits the T-cell receptor (TCR) activity, which prevents T-cells from proliferating [11]. Second, when PD-L1 binds to PD-1 on T-cells, it prevents the production of cytokines necessary for T-cell function, like interferon-gamma (IFN-γ) and tumor necrosis factor-alpha (TNF-α) [12].

The soluble programmed death-ligand 1 (sPD-L1) protein is released from cells and travels in the bloodstream. It is a fragment of the PD-L1 protein, which is generally expressed in the cell's plasma membrane, on the surface of exosomes, in the cell nucleus, and as a soluble protein that circulates in the blood. T-cell activation and function can be suppressed by sPD-L1 through binding to PD-1 receptors on T-cells. Monoclonal antibodies can effectively stop this interaction, which has significant anticancer effects [13]. The ability of the injected antibody to activate T-cells to kill tumor cells with the help of tumor-infiltrating lymphocytes is critical to the effectiveness of an anti-PD-1/PD-L1 therapy [14]. The induction of PD-L1 expression can occur through the influence of various inflammatory cytokines and IFN-γ. This process is observed as part of the immune response to inflammation, and it plays a significant role in regulating immune activity. The association between the PD-1/PD-L1 checkpoint and inflammatory effects is well established, and it has become increasingly evident that this checkpoint is involved in a range of diseases and conditions beyond cancer [15].

The significance and impact of sPD-L1 expression on clinicopathological characteristics and its predictability are currently under debate among researchers [16-18]. While some studies suggest that PD-L1-expressing HNSCC leads to unfavorable clinical outcomes [17,19], others propose that it results in increased survival [20,21]. Malignant cells from various types of cancer, such as lung, breast, and renal cell carcinomas, have been found to exhibit high levels of PD-L1 expression. This overexpression has been linked to increased aggressiveness and poor prognosis [22]. To assess PD-L1 expression in both tumor tissue and its surrounding microenvironment, a biopsy is typically necessary, although this approach has its limitations. However, there is a promising alternative: soluble serum biomarkers. These biomarkers can provide valuable information about the tumor's status and even predict survival outcomes while using a minimally invasive approach [23,24].

This study aims to examine the potential utility of sPD-L1 as a prognostic indicator for HNSCC patients undergoing concurrent chemotherapy and radiation therapy. Given the limited research on this topic, this investigation explores the feasibility of utilizing sPD-L1 as a biomarker to predict and evaluate outcomes for this patient population.

## Materials and methods

The research was conducted between August 2018 and September 2022, and it was approved by the Ethics Committee of Zagazig University (2018-Aug-226). The study included 106 patients with locally advanced HNSCC. The study included patients under 77 years of age diagnosed with HNSCC through histopathological examination. These patients had stages III to IVA disease, according to the American Joint Committee on Cancer (AJCC) Eighth edition staging system [25], and a performance status (PS) score of 0 or 1 on the Eastern Cooperative Oncology Group (ECOG) scale [26]. They exhibited normal hematological, renal, and hepatic functions. Patients had to sign an informed consent form before joining the study. Exclusion criteria for this study were patients with a history of other malignancies or concurrent malignancies, those with poor PS scores (ECOG: 2, 3), abnormal renal or hepatic functions, and abnormal hematological indices. Additionally, patients with known autoimmune diseases or immunodeficiency, prior treatment for HNSCC (such as surgery, radiation therapy, or chemotherapy), severe comorbidities that could significantly impact prognosis or tolerance to concurrent chemotherapy and radiation therapy, pregnancy or breastfeeding, inability to provide informed consent or comply with study requirements, and distant metastasis were also excluded.

The initial assessment involved a thorough medical history and physical examination, a computed tomography (CT) scan, or magnetic resonance imaging (MRI) of the head and neck. In cases where there were indications of potential bone-related symptoms, a bone scan was also included in the evaluation [27]. A triple endoscopy was carried out to assess the tumor's size and whether it had spread to surrounding regions.

Plan of treatment

The treatment plan consisted of three courses of induction chemotherapy using docetaxel-cisplatin-fluorouracil (TPF). After evaluating the response to treatment, concurrent chemoradiation (CCRT) was given to the patients. Treatment was carried out at the Clinical Oncology Department, Zagazig University. Depending on the results, organ preservation or surgery was recommended for the primary tumor site, and a lymph node neck dissection was performed on the affected lymph nodes (N2-N3). The clinical response evaluation adhered to the World Health Organization (WHO) guidelines for response assessment [28].

All patients were given definitive curative CCRT based on their disease stage and overall health condition. This means that all patients received neoadjuvant chemotherapy followed by radiotherapy. After 12 weeks of completion of radiotherapy, the response to treatment was assessed. During the first phase of radiation therapy, the primary tumor site and neck received a dose of 44 Gy using a parallel opposed field and a low frontal cervical radiation field. The second phase involved a 22-26 Gy dose administered using an off-cord bilateral parallel opposed field. In some cases, a posterior neck electron boost was given, increasing the dose by 6-12 MeV electrons to the posterior neck area. During the radiation therapy, the patient was given a weekly dosage of 40 mg/m^2^ of cisplatin.

Assessment of sPD-L1

We analyzed sPD-L1 levels in the blood of 106 patients with HNSCC and 60 healthy individuals of matched age and gender as a control. Among the control participants, there were eight females (13.3%). The ages of the control subjects ranged between 36 and 73 years, and 20 (33.33%) subjects were smokers. We used the Thermo Fisher Scientific Invitrogen Human sPD-L1 ELISA Kit (Cat. No. BMS2212, Vienna, Austria). We collected blood samples from patients and healthy controls using standard laboratory procedures and then separated the serum by centrifugation. The serum was then divided into smaller portions and stored at -80 °C until we analyzed by an ELISA reader (Sunrise, Techan Austria GmbH, Grödig, Austria).

Statistical analysis

In this study, we used IBM SPSS Statistics (Version 27.0, IBM Corp., Armonk, NY, USA) for statistical analyses to investigate the association between demographic and clinical factors and sPD-L1 levels in HNSCC patients. We used unpaired t-tests and multiple regression analysis to compare sPD-L1 levels between subgroups and explore the relationship with demographic and clinical factors. We also used an unpaired t-test and receiver operating characteristics (ROC) analysis to examine the diagnostic accuracy of sPD-L1 in distinguishing HNSCC patients from healthy individuals. In addition, we performed survival analysis using the Kaplan-Meier method and Cox proportional hazards regression analysis to evaluate the association between sPD-L1 levels and survival in HNSCC patients.

## Results

Table 1 provides information about patients with HNSCC, including their age, sex, smoking habits, tumor location, tumor grade, ECOG performance status, and clinical stage. The study comprised 106 participants, with 94 males (88.7%) and 12 females (11.3%). Out of the total participants, 70 (66.0%) were smokers, while 36 (34.0%) were nonsmokers. The tumors were located in four areas: the oral cavity, the oropharynx, the hypopharynx, and the larynx. The oropharynx was the most prevalent site of the tumor, with 45 (42.5%) patients having malignancies there. Twenty-seven (25.5%) patients had GI-GII tumors, and 79 (74.5%) had GIII-GIV tumors. The majority of our patients (86, 80.8%) had an ECOG performance status of 0, meaning they could perform all tasks without limitations. The rest of the patients had an ECOG performance status of 1, indicating that they could do most things but had some limitations. The majority of patients (55, 51.9%) had stage III tumors, while 51 (48.1%) had stage IV tumors. The data also include the cancers' T and N stages.

**Table 1:** Demographic and clinical characteristics of the study population G, grade; ECOG, Eastern Cooperative Oncology Group; T, tumor; N, lymph node

Age (years)	
Range	35-75
Mean ± SD	55.45 ± 36.5
Sex, *n* (%)	
Male	94 (88.7)
Female	12 (11.3)
Smoking, *n*​ ​​ (%)	
Smoker	70 (66)
Nonsmoker	36 (34)
Site, *n* ​​​​​​ ​(%)	
Oral cavity	15 (14.2)
Oropharynx	45 (42.5)
Hypopharynx	32 (30.1)
Larynx	14 (13.2)
Tumor grade, *n* (%)	
GI-GII	27 (25.5)
GIII-GIV	79 (74.5)
ECOG performance status, *n* (%)	
0	85 (80.2)
1	21 (19.8)
Clinical stage, *n* (%)	
Stage III	55 (51.9)
T2N1	16 (15.1)
T3N0	21 (19.8)
T3N1	18 (17)
Stage IV	51 (48.1)
T1N2	4 (7.8)
T2N2	6 (11.8)
T3N2	6 (11.8)
T3N3	4 (7.8)
T4N0	8 (15.7)
T4N1	5 (9.8)
T4N2	14 (27.5)
T4N3	4 (7.8)

Table 2 presents data on the response rates of patients with locally advanced HNSCC. It outlines the number and percentage of patients considered for evaluating their response following induction chemotherapy, concurrent chemoradiotherapy, and completion of all therapy. Additionally, the table displays the number and percentage of patients who achieved a complete response (CR), partial response (PR), stable disease, or progressive disease (PD) during each phase of treatment. The data pertain to patients who were considered for response evaluation. After induction chemotherapy, two patients were excluded from the study due to irregular follow-up: 35 patients (33%) achieved a CR, 60 patients (56.6%) achieved a PR, seven patients (6.6%) had stable disease, and two patients (1.9%) had PD. Following concurrent chemoradiotherapy, 75 patients (72.2%) achieved a CR, 25 patients (24.1%) achieved a PR, three patients (2.8%) had stable disease, and one patient (0.9%) had PD. After the completion of all therapy, 84 patients (80.77%) achieved a CR, 16 patients (15.38%) achieved a PR, three patients (2.89%) had stable disease, and one patient (0.96%) had PD.

**Figure 1:**
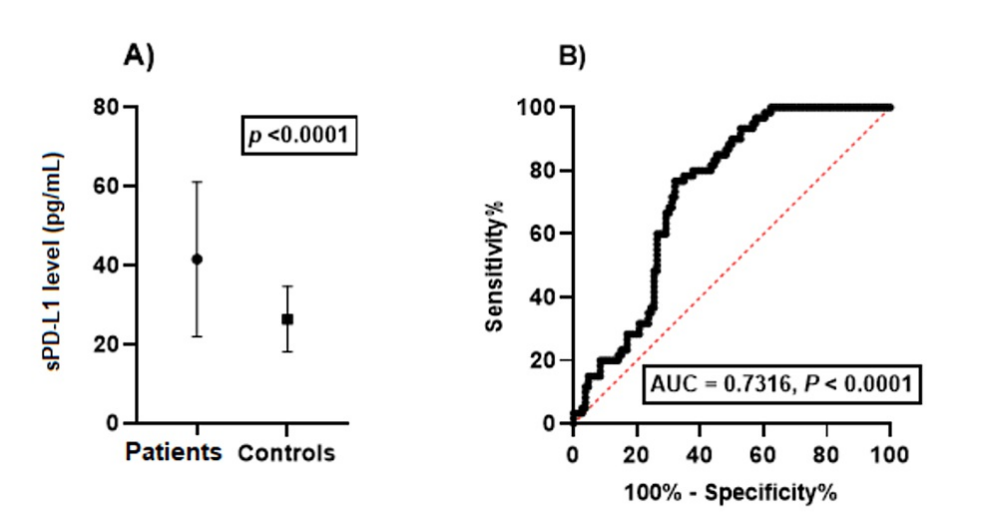
sPD-L1 levels in HNSCC patients and controls. (A) Results of an unpaired t-test comparing the sPD-L1 levels in the serum of patients with HNSCC and controls. The results of the t-test show a *P*-value <0.0001. (B) ROC analysis to evaluate the accuracy of sPD-L1 in distinguishing patients with HNSCC from healthy individuals. ROC, receiver operating characteristics; sPD-L1, soluble programmed death-ligand 1; HNSCC, head and neck squamous cell carcinoma; AUC, area under the curve

Figure 1A displays the outcomes of an unpaired t-test that compared sPD-L1 levels in the serum of HNSCC patients and controls. The results indicate a significant difference between the two groups, with a P-value of less than 0.0001. The calculated t-value is 5.717, and the degree of freedom (df) is 164. These results are considered statistically significant.

**Table 2:** Response rates to induction chemotherapy, concurrent chemoradiotherapy, and completion of all therapy in the study population. CR, complete response; PR, partial response; PD, progressive disease

Response to treatment	Number	%
Evaluable for a response after induction chemotherapy	106	100
CR	35	33
PR	60	56.6
Stable disease	7	6.6
PD	2	1.9
Excluded patients	2	1.9
Evaluable for a response after concurrent chemoradiotherapy	104	100
CR	75	72.2
PR	25	24.1
Stable disease	3	2.8
PD	1	0.9
Evaluable for response at completion of all therapy	104	100
CR	84	80.77
PR	16	15.38
Stable disease	3	2.89
PD	1	0.96

We analyzed the ROC curve to evaluate the accuracy of a test in distinguishing patients with HNSCC from healthy individuals based on their serum PD-L1 levels (Figure 1B). The analysis showed a moderate level of accuracy with an area under the ROC curve of 0.7316. The 95% confidence interval for the area ranged from 0.6570 to 0.8062, with a standard error of 0.03807. A statistically significant *P*-value of less than 0.00001 indicates that the test can differentiate between the two groups based on their serum PD-L1 levels to some degree.

The cutoff value for this analysis was 31.51 pg/mL. This threshold yielded a sensitivity of 76.67% and a specificity of 66.06%.

In this study, we examined the connection between demographic and clinical data and sPD-L1 levels (Table 3). The table shows the mean and standard deviation (SD) of sPD-L1 levels for each subgroup, as well as the results of a t-test and the corresponding *P*-value, which were used to find subgroups with significantly different sPD-L1 levels.

**Table 3:** Correlation between demographic and clinical variables and sPD-L1 levels in HNSCC patients. HNSCC, head and neck squamous cell carcinoma; sPD-L1, soluble programmed death-ligand 1; SD, standard deviation; T, tumor; N, lymph node; TNM, tumor, lymph node, metastasis

Age (years)	*n* (%)	sPD-L1 (Mean ± SD)	*t*-Test	P
35-45	64 (60.4)	53.80 ± 14.95	12.62	<0.0001
46-75	42 (39.6)	22.85 ± 6.586		
Gender				
Male	94 (88.7)	44.82 ± 18.30	5.47	<0.0001
Female	12 (11.3)	15.79 ± 1.728		
Smoking				
Smoker	70 (66)	52.05 ± 15.40	11.68	<0.0001
Nonsmoker	36 (34)	21.09 ± 5.337		
Tumor grade				
I-II	27 (25.5)	18.47 ± 3.006	9.795	<0.0001
III-IV	79 (74.5)	49.42 ± 16.27		
T-stage				
T1-T2	24 (22.6)	17.86 ± 2.567	8.914	<0.0001
T3-T4	82 (77.4)	48.47 ± 16.71		
N-stage				
N0-N1	24	29.54± 13.94	3.085	0.0027
N2-N3	74	41.50± 17.22		
TNM stage				
Stage III	55 (51.9)	40.96 ± 14.36	2.711	0.0078
Stage IV	51 (48.1)	50.84 ± 22.52		

This table presents information on the age groups of the study population and the number of patients in each group. The study found that patients aged 35 to 45 years had significantly higher sPD-L1 levels (mean ± SD = 53.80 ± 14.95) compared to patients aged 46 to 75 years (mean ± SD = 22.85 ± 6.586), with a *P*-value <0.0001.

Males had significantly higher sPD-L1 levels (mean ± SD = 44.82 ± 18.30) than females (mean ± SD = 15.79 ± 1.728), with a *P*-value <0.0001. In the study population, smokers had higher sPD-L1 levels (mean ± SD = 52.05 ± 15.40) compared to nonsmokers (mean ± SD = 21.09 ± 5.337). This difference was statistically significant with a *P*-value <0.0001.

Patients with higher grade tumors (GIII-GIV) have significantly higher sPD-L1 levels (mean ± SD = 49.42 ± 16.27) compared to those with lower grade tumors (GI-GII) (mean ± SD = 18.47 ± 3.006) with a *P*-value <0.0001.

Patients with advanced T-stage tumors (T3-T4) have significantly higher sPD-L1 levels (mean ± SD = 48.47 ± 16.71) compared to those with early-stage T tumors (T1-T2) (mean ± SD = 17.86 ± 2.567) with a *P*-value <0.0001.

There is a difference in sPD-L1 levels between patients with advanced N-stage tumors (N2-N3) and early-stage N tumors (N0-N1). Patients with advanced tumors had significantly higher levels (mean ± SD = 41.50 ± 17.22) compared to those with early-stage tumors (mean ± SD = 29.54 ± 13.94) with a *P*-value of 0.0027.

The TNM stage of advanced tumors (stage IV) had significantly higher sPD-L1 levels (mean ± SD = 50.84 ± 22.52) compared to early-stage tumors (stage III) (mean ± SD = 40.96 ± 14.36) with a *P*-value of 0.0078.

Table 4 shows the results of a multiple regression analysis of sPD-L1 in patients with HNSCC. The study examined various independent variables, including grade, lymph nodes, TNM stage, sex, age, smoking, tumor site, and tumor size. The dependent variable was sPD-L1. The estimate for the intercept is 0.002413, and the confidence interval indicates that the intercept is not significantly different from zero. Therefore, it is not a significant predictor of sPD-L1.

**Table 4:** Multiple regression analysis of sPD-L1 in patients with head and neck squamous cell carcinoma. T, tumor; N, lymph node; TNM, tumor, lymph node, metastasis; ns, nonsignificant; sPD-L1, soluble programmed death-ligand 1; hs, highly significant.

Variable	Estimate	Standard error	95% confidence interval	|t|-test	*P*-value	*P*-value summary
Intercept	0.002413	0.1284	-0.2527 to 0.2575	0.01879	0.985	ns
Grade	0.0002234	0.04909	-0.09731 to 0.09776	0.004551	0.9964	ns
N	0.002879	0.05453	-0.1055 to 0.1112	0.05279	0.958	ns
TNM stage	-0.0001532	0.04676	-0.09306 to 0.09276	0.003276	0.9974	ns
Sex	0.002949	0.05301	-0.1024 to 0.1083	0.05563	0.9558	ns
Age	0.8378	0.04508	0.7482 to 0.9274	18.59	<0.0001	hs
Smoking	0.1618	0.04667	0.06902 to 0.2545	3.466	0.0008	hs
Site	-0.002949	0.009096	-0.02102 to 0.01512	0.3242	0.7465	ns
T	-0.002081	0.04075	-0.08305 to 0.07889	0.05106	0.9594	ns

The coefficients and confidence intervals for the other variables, including histopathological tumor grade, lymph nodes N, TNM stage, sex, primary tumor site, and tumor size, were also close to zero. This suggests that these variables are not significant predictors of sPD-L1.

However, age and smoking had *P*-values <0.05, indicating that they are significantly associated with sPD-L1. Age significantly affected sPD-L1, with a *P*-value <0.0001. Similarly, smoking also had a significant effect on sPD-L1, with a *P*-value of 0.0008.

**Figure 2:**
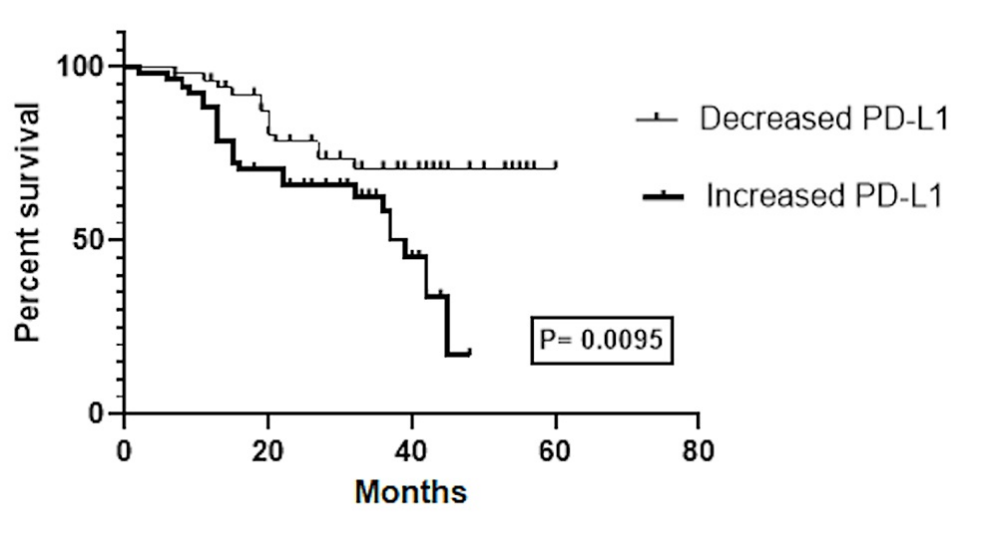
The association between sPD-L1 levels and survival in HNSCC patients. The survival analysis showed a significant difference in the survival rates of patients with raised and decreased sPD-L1 levels. Patients with lower sPD-L1 levels did not achieve their median survival time, indicating that patients with greater sPD-L1 levels may have a worse prognosis. sPD-L1, soluble programmed death-ligand 1; HNSCC, head and neck squamous cell carcinoma

Our study's survival analysis revealed a notable contrast in the survival outcomes between patients with reduced and elevated sPD-L1 levels. The log-rank (Mantel-Cox) test computed a chi-square value of 6.725 and a *P*-value of 0.0095, indicating a significant difference in the survival curves. Patients with increased sPD-L1 levels had a median survival time of 39 months, while those with decreased sPD-L1 levels did not reach their median survival time, suggesting that patients with higher sPD-L1 levels may have a poorer prognosis (Figure 2).

## Discussion

HNSCC is a prevalent malignancy that arises from the epithelial cells lining the interior surfaces of the head and neck. This type of cancer accounts for about 90% of all head and neck cancer cases [29]. Traditionally, surgery, radiation therapy, and chemotherapy were the main ways to treat HNSCC. However, recent advances in immunotherapy have created new possibilities for treating this disease [24]. PD-L1 is a protein that suppresses the immune response by binding to its corresponding receptor, PD-1, on T-cells' surfaces. Essentially, this interaction acts as a brake on the immune system, preventing it from attacking healthy cells [30]. Recent studies have demonstrated that elevated levels of PD-L1 expression in HNSCC tumors facilitate cancer cells in evading the immune system, thereby enabling unchecked proliferation. However, these same tumors exhibit a higher propensity to respond positively to immunotherapy drugs that target the PD-L1/PD-1 pathway. PD-L1 enables cancer cells to evade the immune system's detection and reproduce unencumbered. Nonetheless, recent studies have revealed that HNSCC tumors exhibiting heightened levels of PD-L1 expression are more prone to react favorably to immunotherapy medications that directly aim at the PD-L1/PD-1 pathway [18].

The current study aims to evaluate the feasibility of utilizing sPD-L1 as a prognostic biomarker for patients diagnosed with HNSCC undergoing concurrent chemotherapy and radiotherapy. The main objective of this investigation is to explore the potential of sPD-L1 as a prognostic factor for this cohort, despite the limited amount of comprehensive research in this field.

According to our study, the majority of the patients with HNSCC (94, 88.7%) were male, which aligns with previous studies showing that men are more likely to develop this type of cancer [31]. Smoking was also a significant risk factor, as 66% (70) of our patients had a smoking history, consistent with previous research [32]. Based on previous research, the most frequent location for HNSCC is the oropharynx, followed by the oral cavity, the hypopharynx, and the larynx. This aligns with the current study's findings, which also identified the oropharynx as the most common site for the tumor [33,34]. Our research shows that most patients with HNSCC have advanced-stage cancer, with stage III or IV being the most prevalent. This indicates the aggressive nature of the disease and the difficulty of detecting it early. Notably, despite their condition, most patients had an ECOG performance status of 0, indicating that it did not significantly impact their daily activities [35]. Our study found that most patients had GIII-GIV tumors, which typically indicate a more aggressive disease and a poor prognosis [36]. Our research aligns with previous studies, indicating the necessity for further investigation into successful prevention and treatment methods. In addition, our research emphasizes the importance of identifying risk factors and detecting problems early to improve outcomes for patients. Our research shows that patients with locally advanced HNSCC respond well to treatment. Our data reveal that 95 (89.6%) patients had either a complete or partial response with induction chemotherapy, while 102 (96.3%) had similar outcomes with concurrent chemoradiotherapy. Moreover, 99 (96.15%) patients attained a complete or partial response after the entire treatment. Our research supports the use of neoadjuvant chemotherapy followed by CCRT as an effective treatment option for managing locally advanced HNSCC. This study confirms that chemoradiotherapy is an effective treatment for patients with locally advanced HNSCC, which is consistent with previous research in this field [37,38]. Additionally, the number of patients who achieved complete remission is promising, as this has been linked to better survival rates [39,40]. It is crucial to remember that this information only refers to one institution's experience with locally advanced HNSCC and that a larger trial should confirm it. Additionally, the sample size is small, which may reduce the statistical power of the findings. Our results suggest that individuals with locally advanced HNSCC could benefit from induction chemotherapy and concomitant chemoradiotherapy as effective treatment options.

The current research has found a notable difference in the levels of serum sPD-L1 between healthy individuals and those with HNSCC. This is in line with previous studies that have shown higher levels of PD-L1 in the tumor microenvironment of HNSCC patients [41].

In their study, Yi et al. emphasized the importance of monitoring sPD-L1 levels in NSCLC patients undergoing anti-PD-1 immunotherapy. Their study indicates that observing changes in sPD-L1 levels and IL-8 can offer valuable information on the patient's response to treatment. This technique can serve as a valuable tool in evaluating the effectiveness of anti-PD-1 immunotherapy in NSCLC patients [42].

According to our findings, serum sPD-L1 levels can distinguish between HNSCC patients and healthy people with moderate accuracy. Based on the area under the ROC curve (AUC) of 0.7316, the test can differentiate between the two groups. The AUC measures the overall performance of a binary classifier, and an AUC score of 0.7316 is considered moderate. The AUC score of 0.7316 suggests that the test can be valuable, but it may not be enough to serve as the only diagnostic tool. The test's accuracy was evaluated at a cutoff value of 31.51 pg/mL, resulting in a sensitivity of 76.67% and specificity of 66.06%. This means the test correctly identifies 76.67% (81.27) of patients with HNSCC while excluding 66.06% (70.02) of healthy individuals. However, the test's lower specificity poses a risk of false positives, which could result in unnecessary diagnostic procedures. The study indicates that serum sPD-L1 levels could be a valuable biomarker for diagnosing and managing HNSCC. However, more research is required to verify the accuracy of the test and its usefulness in clinical applications.

Our research showed a significant correlation between sPD-L1 levels and the demographic and clinical characteristics of patients with HNSCC. This indicates that sPD-L1 could be a valuable biomarker for HNSCC, and it also sheds light on the factors that can affect sPD-L1 levels in this patient group. Several research studies have explored the correlation between sPD-L1 levels and clinical factors in individuals with HNSCC. Research by Ancel et al. has shown that high sPD-L1 levels in patients with non-small-cell lung cancer are associated with advanced stages of the disease and worse survival rates [43]. Similarly, Xu et al. found that patients with advanced solid tumors tend to have higher levels of PD-L1 [44]. We discovered that patients aged between 35 and 45 years had significantly higher levels of sPD-L1 than those aged between 46 and 75 years. This interesting finding aligns with the research of Molga-Magusiak et al., who also found higher sPD-L1 levels in younger HNSCC patients [45]. However, the reasons for this correlation are still unknown. The results of our study revealed that male HNSCC patients and smokers have higher levels of sPD-L1 than female HNSCC patients and nonsmokers, respectively. This finding is consistent with previous research, which has shown that males and smokers are at a higher risk of developing HNSCC and that smoking may play a role in modulating the immune response to HNSCC [46]. Previous studies, such as Zhang et al., have also found a correlation between sPD-L1 levels and tumor stage and grade [47]. This supports our observation that patients with more advanced tumors and higher grades tend to have higher sPD-L1 levels. The findings suggest that measuring sPD-L1 levels can assist in detecting HNSCC and that factors like age and medical history can influence these levels. However, further research is needed to validate these findings and assess the potential of sPD-L1 as a biomarker for HNSCC.

Our research showed that age and smoking significantly affect sPD-L1 levels in patients with HNSCC. However, other factors such as tumor grade, lymph nodes, TNM stage, sex, primary tumor site, and tumor size do not have a significant impact. Several studies have explored how sPD-L1 levels relate to demographic and clinical factors among HNSCC patients. In particular, the study by Zhang et al. revealed that high sPD-L1 levels were linked to advanced disease stages, lymph node metastasis, and worse survival outcomes in HNSCC patients [47]. Similarly, a study by Molga-Magusiak et al. reported that sPD-L1 levels were higher in HNSCC patients with advanced disease stages and lymph node metastasis [45].

Research indicates that younger patients with HNSCC are likely to have higher levels of sPD-L1 than older patients, as age is a significant predictor of sPD-L1 levels. This aligns with previous studies that have identified age as a risk factor for HNSCC and have also found higher sPD-L1 levels in young patients with other types of cancer, including lung, hypopharyngeal, and esophageal cancers [48-50]. Although not entirely clear, the connection between age-related changes in the immune system and the tumor microenvironment may be responsible for this correlation [49].

Our study's survival analysis offers crucial insights into the potential prognostic value of sPD-L1 levels in HNSCC. The log-rank test showed that the survival rates of patients with low sPD-L1 levels significantly differed from those with high sPD-L1 levels. This suggests that sPD-L1 levels could be a valuable biomarker for predicting survival outcomes in HNSCC patients. Our research study indicates that patients with higher levels of PD-L1 may experience a poorer prognosis than those with lower levels. The study found that patients with increased sPD-L1 levels had a median survival time of 39 months, whereas those with decreased levels did not reach their median survival time. This finding is consistent with other studies that have shown a connection between PD-L1 expression and survival rates in various types of cancer, including HNSCC [51-54]. However, one potential limitation of our study is the relatively modest sample size of 106 patients. Increasing the sample size could enhance the reliability and applicability of the findings.

## Conclusions

In conclusion, our study highlights the potential of sPD-L1 as a prognostic biomarker for HNSCC patients undergoing concurrent chemotherapy and radiotherapy. The study found that higher levels of sPD-L1 were associated with poorer survival outcomes, indicating that sPD-L1 could be a valuable biomarker for predicting survival in HNSCC patients. Furthermore, patients with lower levels of sPD-L1 may have better chances of surviving, as shown by the noticeable difference in the survival rates between those with decreased and increased levels. These findings have important implications for predicting survival outcomes and guiding treatment decisions in HNSCC patients. However, further studies are needed to validate these findings and determine the clinical utility of sPD-L1 as a prognostic biomarker for HNSCC. Overall, our study contributes to the growing body of evidence supporting the role of sPD-L1 levels in managing HNSCC.
